# Impact of fibrinogen concentrate alone or with prothrombin complex concentrate (+/− fresh frozen plasma) on plasma fibrinogen level and fibrin-based clot strength (FIBTEM) in major trauma: a retrospective study

**DOI:** 10.1186/1757-7241-21-74

**Published:** 2013-10-08

**Authors:** Christoph J Schlimp, Wolfgang Voelckel, Kenji Inaba, Marc Maegele, Herbert Schöchl

**Affiliations:** 1 Ludwig Boltzmann Institute of Experimental and Clinical Traumatology, AUVA Research Centre, Vienna, Austria; 2 Department of Anaesthesiology and Intensive Care Medicine, AUVA Trauma Centre, Salzburg, Austria; 3 Department of Surgery, Los Angeles County and University of Southern California Medical Center, Los Angeles, CA, USA; 4 Department of Trauma and Orthopedic Surgery, University of Witten/Herdecke, Cologne-Merheim Medical Center (CMMC), Cologne, Germany

**Keywords:** Fibrinogen concentrate, Prothrombin complex concentrate, Fresh frozen plasma, Thromboelastometry (ROTEM)

## Abstract

**Background:**

Low plasma fibrinogen concentration is a predictor of poor outcome in major trauma patients. The role of fibrinogen concentrate for rapidly increasing fibrinogen plasma levels in severe trauma is not well defined.

**Methods:**

In this retrospective study we included severe trauma patients treated with fibrinogen concentrate alone (FC group), fibrinogen concentrate with prothrombin complex concentrate (FC–PCC group) or fibrinogen concentrate with PCC and fresh frozen plasma (FC–PCC–FFP group). PCC was generally administered as the second step of intraoperative therapy, while FFP was only administered as a third step. All patients received ≥1 g fibrinogen concentrate within 24 hours. Plasma fibrinogen concentration and ROTEM parameters upon emergency room (ER) admission, intensive care unit (ICU) admission, and after 24 hours were analysed.

**Results:**

Among 157 patients fulfilling the inclusion criteria, 83% were male; mean age was 44 years and median injury severity score (ISS) was 29. Standard coagulation tests reflected increasing severity of coagulopathy with increasing complexity of haemostatic therapy (highest severity in the FC–PCC–FFP group; p < 0.0001). Total 24-hour fibrinogen concentrate dose also increased with complexity of haemostatic therapy. Plasma fibrinogen concentration was maintained, with no significant difference between ER admission and ICU admission in all patient groups. FIBTEM clot firmness at 10 minutes (CA_10_) was similarly maintained, albeit with a small increase in the FC–PCC group. Fibrinogen concentration and FIBTEM CA_10_ were within the normal range in all groups at 24 hours. The ratio of fibrinogen concentrate to red blood cells (g:U) ranged between 0.7:1.0 and 1.0:1.0.

**Conclusion:**

Fibrinogen concentrate therapy maintained fibrinogen concentration and FIBTEM CA_10_ during the initial phase of trauma care until ICU admission. After 24 hours, these parameters were comparable between the three groups and within the normal range for each of them. Further studies are warranted to investigate the effect of fibrinogen concentrate on clinical outcomes.

## Background

In-hospital exsanguination has been reported in 26–39% of all casualties in civilian trauma centres [1,2]. Moreover, it is estimated that up to 20% of trauma-associated deaths are potentially preventable if early and rapid control of blood loss and coagulopathy could be established [3].

Recent data from military and civilian trauma centres revealed that early and high-volume fresh frozen plasma (FFP) transfusion is associated with a favourable survival rate [4,5]. However, several important shortcomings of FFP transfusion should be considered. Firstly, for immediate treatment pre-thawed FFP must be available. Outside busy trauma centres with high patient throughput this is hard to establish. Secondly, coagulation factors do not decrease uniformly during major bleeding. Fibrinogen has been identified as the most vulnerable coagulation protein, being the first to reach critically low levels [6]. This protein is required for both platelet aggregation and fibrin formation [7]. Fibrinogen supplementation may also compensate for dilutional coagulopathy or impaired haemostasis due to thrombocytopenia [8,9] Low plasma fibrinogen concentration is common among major trauma patients and associated with poor clinical outcomes [10-13]. Importantly, FFP and solvent/detergent-treated plasma (SD-plasma) contain only low levels of fibrinogen [14]. Therefore, rapid normalization or even maintenance of plasma fibrinogen concentration in severe bleeding trauma patients is difficult when using plasma alone [10,15]. Pre-thawed or freeze dried (lyophilized) plasma can be provided much faster, but at present their availability is limited and both have a similarly low concentration of fibrinogen. Cryoprecipitate and purified human fibrinogen concentrate represent potential alternatives to plasma for maintaining or increasing the patient’s fibrinogen level, subject to these products’ availability. Cryoprecipitate has to be pooled (often just before administration); it also contains variable amounts of fibrinogen, has to be thawed before use and is not virus inactivated [16,17]. In contrast, fibrinogen concentrate contains a well-defined concentration of fibrinogen, has a good safety profile and is immediately available for use [18]. A recent randomized, placebo-controlled study in complex cardiac surgery revealed a significant reduction in allogeneic blood transfusion in patients treated with fibrinogen concentrate [19]. Several retrospective studies and case series have reported improved outcomes using fibrinogen concentrate in trauma [20-26].

Despite common use of fibrinogen concentrate in many European trauma centres, little is known about its effect on plasma fibrinogen concentration [21]. We retrospectively investigated major trauma patients presenting at the AUVA Trauma Centre, Salzburg who were treated with fibrinogen concentrate as first-line coagulation therapy. The aim was to compare plasma fibrinogen concentration and FIBTEM clot amplitude at 10 minutes’ running time (CA_10_) upon admission to the emergency room (ER), admission to the intensive care unit (ICU) and 24 hours after ER admission. According to haemostatic treatment administered, three groups of patients were investigated: i. FC group, those receiving fibrinogen concentrate only, ii. FC–PCC group, those receiving fibrinogen concentrate and prothrombin complex concentrate (PCC) and iii. FC–PCC–FFP group, those receiving fibrinogen concentrate, PCC and FFP.

## Methods

Following local ethics committee approval (415-EP/73/197-2013) we performed a retrospective analysis of major trauma patients admitted to the AUVA Trauma Centre Salzburg between 2010 and 2012. The inclusion criterion was administration of fibrinogen concentrate as first-line coagulation therapy, which also included patients who additionally received PCC and FFP. Exclusion criteria were burns, pregnancy and participation in other studies. Patients receiving anticoagulation medication pre-trauma were not excluded from the study as this information is not always known when a patient arrives at the ER. Patients are usually treated with a ROTEM guided algorithm, receiving fibrinogen concentrate and PCC according to their actual needs.

Demographic data, laboratory data, trauma scores such as injury severity score (ISS), new injury severity score (NISS), Glasgow coma scale (GCS) and outcomes data were obtained from the electronic trauma database.

Blood samples were drawn as soon as possible following ER admission, either from arterial lines or, in less severe cases, from venous lines. For coagulation monitoring, citrated blood was collected in two standard coagulation tubes. Viscoelastic testing was run within minutes on a ROTEM analyser in the ER. The following tests were performed: an extrinsically activated test using tissue factor (EXTEM) and a fibrin polymerization test (FIBTEM) which inhibits platelets’ contribution to clot elasticity. The following parameters were investigated: EXTEM clotting time (CT, normal range 38–79 seconds), EXTEM clot formation time (CFT, normal range 34–159 seconds), EXTEM alpha angle (normal range 63–83°), EXTEM maximum clot firmness (MCF, normal range 50–72 mm) and FIBTEM CA_10_ (normal range 7–23 mm).

A second citrated blood sample was drawn for standard coagulation tests (SCTs) in the central laboratory. The following parameters were assessed after centrifugation of the sample: fibrinogen concentration (Clauss Method, normal range 200–450 mg/dL), prothrombin time (PT, normal range 11.0–16.1 seconds) and activated partial thromboplastin time (aPTT, normal range 23.7–34.9 seconds).

Blood cell count and blood gas analyses were performed upon ER admission and ICU admission; haemoglobin (Hb, normal range 13.0–17.7 g/dL) and platelet count (normal range 150–350,000/μL) were also assessed. ROTEM analyses or SCTs were performed upon ICU admission for patients with suspected coagulopathy. We used the timepoint “ICU” to describe assessments made at the end of surgery or during the first few hours after ICU admission.

SCTs were also run every morning at 7 am and, depending on the patient’s condition, at 4 pm. For the study, we applied the timepoint “24 hours” to describe analyses performed at either 7 am or 4 pm, depending which was closer to 24 hours after hospital admission.

Patients were treated according to our institutional algorithm which was published recently [27]. Briefly, the indication for fibrinogen concentrate is to increase FIBTEM CA_10_ to 10–12 mm for patients with low FIBTEM CA_10_ (<7 mm). Values below 7 mm indicate reduced fibrin polymerization, often resulting from a low plasma fibrinogen level. Fibrinogen concentrate (Haemocomplettan® P; CSL Behring, Marburg, Germany) is then administered, at a dose of 2–6 g (2–4 g if initial FIBTEM CA_10_ 4–6 mm; 6 g if initial FIBTEM CA_10_ 0–3 mm). Platelet concentrate is transfused in patients whose EXTEM CA_10_ remains low (<40 mm) after increasing FIBTEM CA_10_ to 10–12 mm. The algorithm also recommends considering PCC if EXTEM CT is >80 seconds after raising FIBTEM CA_10_ to 10–12 mm, or if EXTEM CA_10_ <30 mm [27]. Prothromplex® (Baxter, Vienna, Austria) was used in most cases. According to our algorithm, tranexamic acid (TXA) should be given if the patient is in shock or if ISS is greater than 15 [27]. However, TXA was not included in the algorithm until 2012, meaning that most of the patients included in this study did not receive TXA and that the use of this product increased during the observation period. Coagulation therapy data were obtained from the anaesthesia records and ICU medical charts. FFP was used mainly on individual discretion of the attending anaesthetist, particularly in cases of ongoing major bleeding, based on the consideration that factors not present in PCC e.g., factor V are missing in the late stages of a major trauma.

The target haemoglobin concentration during the initial operative procedure was 10 g/dL. In the postoperative phase, lower levels of 7–8 g/dL were accepted, depending on the hemodynamic condition of the patient. All transfused allogeneic blood products were recorded in a database (DataLab, Bartels, Graz, Austria), in accordance with standard practice at our centre. Additionally, the amount of cell-saver blood administered was obtained from anaesthesia charts. Each 250 mL of cell-saver blood transfused was considered equivalent to 1 unit of red blood cells (RBCs).

Mortality was defined as death within 30 days of ER admission.

### Statistical analysis

The primary endpoint in this study was plasma fibrinogen concentration and fibrin clot parameters on ICU admission. For all parameters, normality of the data distribution was tested using the Kolmogorov-Smirnov test. Normally distributed parameters were reported as mean ± standard deviation, and those with non-normal distribution were expressed as median and interquartile range (IQR; 25th percentile – 75th percentile). For categorical variables, p-values were derived from Fisher´s exact or the chi-square test. For continuous variables including those with time dependency, between-group differences were analysed using analysis of variance (ANOVA) and the Newman-Keuls test (comparisons of three groups) or the Mann–Whitney *U* test (comparisons of two groups). For within-group comparison of timepoints, the t-test or Mann–Whitney *U* test was used.

Data were analysed for all patients with a value at the timepoint of interest (e.g. patients who died within 24 hours were not included in 24-hour outcomes). Statistical calculations were performed using GraphPad Prism 5.03 (GraphPad Software, La Jolla, CA, USA). The level of significance was set at p < 0.05.

## Results

Between January 2010 and December 2012, 160 patients received fibrinogen concentrate as first-line coagulation therapy within 24 hours of hospital admission. Three patients were excluded because they were enrolled in other studies. Therefore, 157 patients were eligible for further analyses.

The study population comprised 130 (83%) male and 27 (17%) female patients with a mean age of 44 ± 19 years. The median ISS was 29 (23–41), median NISS was 34 (27–47) and median GCS was 11 (5–15). Immediate operative intervention was performed in 139 (89%) patients. Median ICU length of stay was 14 (5–23) days and median hospital length of stay was 26 (14–40) days.

**Figure 1:**
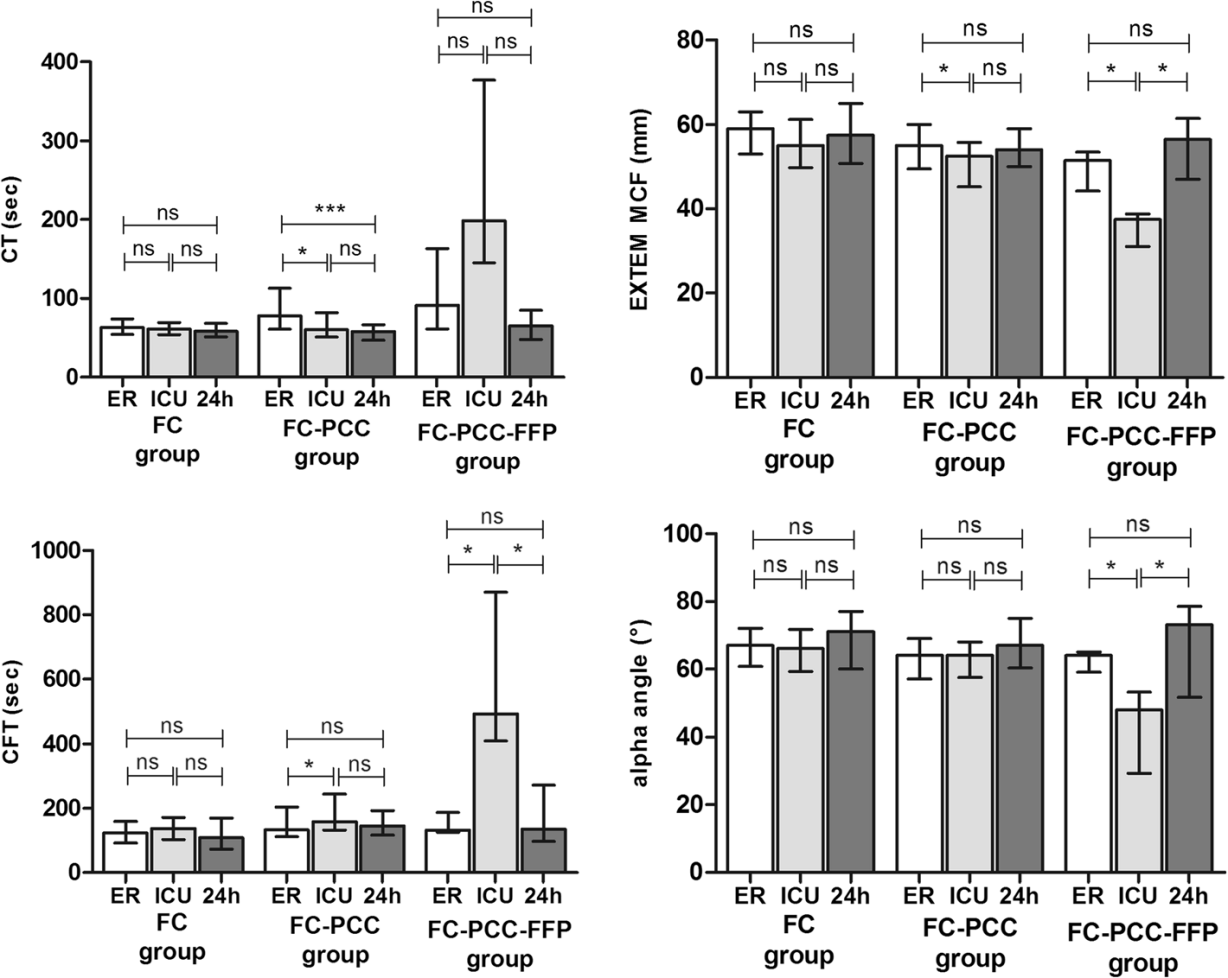
**EXTEM test results.** Results from the extrinsically activated EXTEM assay, performed using ROTEM. Results were obtained at the following timepoints: upon emergency room (ER) admission, intensive care unit (ICU) admission and 24 hours after ER admission. Data are presented as median values; error bars represent interquartile ranges. CT, clotting time; CFT, clot formation time; MCF, maximum clot firmness. FC group, patients receiving fibrinogen concentrate only; FC–PCC group, patients receiving fibrinogen concentrate and prothrombin complex concentrate; FC–PCC–FFP group, patients receiving fibrinogen concentrate, prothrombin complex concentrate and fresh frozen plasma. *p < 0.05, ***p < 0.001, ns = not significant (unpaired *t*-test or Mann–Whitney *U* test).

There were 85 patients in the FC group, 63 in the FC-PCC group and 9 in the FC–PCC–FFP group (Table 1). There were significant between-group differences in ISS and NISS, with highest severity in the FC–PCC–FFP group (p < 0.0001). Laboratory data for the three timepoints are shown in Table 2. Significant between-group differences were observed upon ER admission and ICU admission in platelet count, PT, aPTT and fibrinogen concentration, demonstrating worse coagulation status in the FC–PCC group versus the FC group and worst status in the FC–PCC–FFP group (Table 2). At 24 hours, significant between-group differences were observed in platelet count and aPTT, but there were no significant between-group differences in most other parameters. ROTEM data (EXTEM assay) demonstrated less pronounced between-group differences (Figure 1). The most pronounced findings were prolonged CT and CFT in the FC–PCC–FFP group upon ICU admission, as well as low EXTEM MCF and reduced alpha angle in the FC–PCC–FFP group at the same timepoint. Severe coagulopathy in the FC–PCC–FFP group was therefore demonstrated by EXTEM in a similar manner to the laboratory data. Also similar to the laboratory data, between-group differences in all EXTEM parameters reached statistical significance upon ER admission and ICU admission but not at 24 hours.

**Table 1:** Clinical data upon emergency room (ER) admission of patients receiving fibrinogen concentrate (FC)

	**FC group (n = 85)**	**FC–PCC group (n = 63)**	**FC–PCC–FFP group (n = 9)**	**p-value**
**Age (years)**	40 (27–58)	45 (26–57)	49 (29–58)	0.99
**Age >55 years, n (%)**	24 (28%)	17 (27%)	2 (22%)	0.93
**Male, n (%)**	73 (86%)	51 (81%)	6 (67%)	0.31
**Systolic BP (mmHg)**	110 (90–131)	88 (75–125)	83 (75–105)	0.0068
**Systolic BP ≤90 mmHg , n (%)**	23 (27%)	32 (31%)	5 (56%)	0.0073
**Temperature (°C)**	35.2 (34.1–36.0)	35.7 (34.0–36.35)	35.9 (35.2–36.4)	0.39
**ISS**	27 (20–34)	34 (26–43)	50 (42–58)	<0.0001
**NISS**	30 (26–41)	38 (28–50)	50 (43–58)	0.0002
**ISS ≥16, n (%)**	76 (89%)	63 (100%)	9 (100%)	0.018
**ISS ≥25, n (%)**	53 (62%)	48 (76%)	9 (100%)	0.025
**GCS**	12 (5–15)	10 (5–15)	7 (4–15)	0.58
**pH**	7.33 (7.28–7.38)	7.28 (7.22–7.35)	7.26 (7.17–7.35)	0.0025
**BD (mmol/L)**	3.7 (1.9–5.8)	5.6 (3.4–8.5)	5.8 (4.1–10.7)	0.0005
**Lactate (mmol/L)**	2.12 (1.56–3.48)	3.10 (2.27–5.45)	7.51 (4.51–8.78)	0.0001
Acute operative intervention*, n (%)	74 (87%)	57 (91%)	9 (100%)	0.45

Data are presented as median (interquartile range) or number (percentage of patients). p-values are derived from the ANOVA or chi-square test; significance level p < 0.05.

*All types of intervention and damage control (e.g. orthopaedic surgery).

BD, base deficit; BP, blood pressure; ISS, injury severity score; NISS, new injury severity score; GCS, Glasgow coma scale.

FC group, patients receiving fibrinogen concentrate only; FC–PCC group, patients receiving fibrinogen concentrate and prothrombin complex concentrate; FC–PCC–FFP group, patients receiving fibrinogen concentrate, prothrombin complex concentrate and fresh frozen plasma.

**Table 2:** Blood cell count and standard coagulation tests

	**FC group**		**FC–PCC group**		**FC–PCC–FFP group**		**p-value**
	**n**		**n**		**n**		
**Hemoglobin (g/dL)**							
ER	85	12.8 (11.2–13.9)	63	11.1 (8.8–12.7)	9	8.4 (6.5–11.6)	<0.0001
ICU	70	8.8 (7.8**–**9.9)	44	9.3 (8.1–10.2)	9	8.0 (7.1–10.0)	0.20
24h	81	8.8 (7.8**–**9.9)	51	9.3 (8.1–10.2)	4	8.0 (7.1–10.0)	0.27
**Platelet count (x 10**^**9**^**/L)**							
ER	85	210 (176–251)	63	181 (141–215)	9	203 (162–295)	0.041
ICU	54	148 (108–179)	41	104 (65–126)	7	58 (35–88)	<0.0001
24h	80	122 (95–162)	51	90 (63–111)	4	58 (37–75)	<0.0001
**PT (seconds)**							
ER	83	14.6 (13.4–15.8)	63	17.0 (15.3–19.3)	9	22.0 (17.7–27.9)	<0.0001
ICU	34	17.5 (15.5–19.6)	31	21.0 (17.1–24.3)	7	28.6 (26.6–44.1)	<0.0001
24h	70	16.2 (15.2–17.8)	48	16.7 (15.6–18.0)	4	16.6 (16.1–16.7)	0.49
**aPTT (seconds)**							
ER	82	28.8 (26.0–31.7)	62	33.2 (28.0–43.5)	9	48.8 (35.3–96.5)	<0.0001
ICU	42	32.7 (29.9–36.4)	31	46.9 (36.5–54.7)	8	180.0 (89.7–180.0)	<0.0001
24h	77	37.7 (33.6–40.4)	49	40.2 (37.2–46.7)	4	45.5 (38.2–49.6)	0.0003
**Fibrinogen (mg/dL)**							
ER	79	198 (156–225)	60	151 (112–199)	9	95 (83–161)	<0.0001
ICU	41	188 (147–219)	32	162 (129–203)	8	119 (82–142)	0.0034
24h	76	266 (236–309)	49	274 (222–316)	4	269 (240–298)	0.96

Data are presented as median (interquartile range) or percentage of patients. p-values are derived from the ANOVA; significance level p < 0.05.

aPTT, activated partial thrombin time; PT, prothrombin time.

FC group, patients receiving fibrinogen concentrate only; FC–PCC group, patients receiving fibrinogen concentrate and prothrombin complex concentrate; FC–PCC–FFP group, patients receiving fibrinogen concentrate, prothrombin complex concentrate and fresh frozen plasma.

Results were obtained at the following timepoints: upon admission to the emergency room (ER), admission to the intensive care unit (ICU) and 24 hours (24h) after ER admission; n = number of patients with available data.

**Figure 2:**
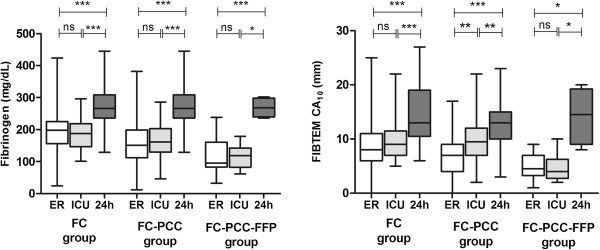
**Fibrinogen concentration and FIBTEM test results.** For the fibrin-based FIBTEM assay, clot amplitude at 10 min running time (CA_10_) is shown. Results were obtained at the following timepoints: upon admission to the emergency room (ER), upon admission to the intensive care unit (ICU) and approximately 24 hours (24h) after ER admission. Data are presented as median values with interquartile ranges; error bars represent minimum and maximum values. FIBTEM, extrinsically activated test of fibrin-based clotting (cytochalasin D inhibits platelet contribution to clot strength); CA_10_, clot amplitude at 10 minutes. FC group, patients receiving fibrinogen concentrate only; FC–PCC group, patients receiving fibrinogen concentrate and prothrombin complex concentrate; FC–PCC–FFP group, patients receiving fibrinogen concentrate, prothrombin complex concentrate and fresh frozen plasma. *p < 0.05, **p < 0.01, ***p < 0.001, ns = not significant (unpaired *t*-test or Mann–Whitney *U* test).

Transfusions of allogeneic blood products and coagulation factor concentrates are shown in Table 3. In general, transfusion requirements were highest in the FC–PCC–FFP group and lowest in the FC group. The median 24-hour dose of fibrinogen concentrate was 3 g (IQR 2–5 g; range 2–11 g) in the FC group, 7 g (IQR 5–10 g; range 2–21 g) in the FC–PCC group and 15 g (IQR 9–17 g; range 6–18 g) in the FC–PCC–FFP group. Thus, the total dose of fibrinogen concentrate differed between groups in a similar manner to ISS, with the highest values for dose and ISS score in the FC–PCC–FFP group and lowest values in the FC group. Plasma fibrinogen level did not change significantly between ER admission and ICU admission in any of the three groups, even though the median dose of fibrinogen concentrate in the FC–PCC group was more than double that in the FC group and median dose in the FC–PCC–FFP group was 4-fold higher than that in the FC group (p < 0.0001 for both comparisons) (Figure 2). FIBTEM CA_10_ increased significantly between ER admission and ICU admission in the FC–PCC group (p = 0.003), but not in either of the other groups. Plasma fibrinogen concentration and FIBTEM CA_10_ were lower in the FC–PCC–FFP group than in the other two groups at ICU admission, despite the high intraoperative dose of fibrinogen concentrate in the FC–PCC–FFP group. No significant between-group differences were observed in either of these parameters at 24 hours and, at this timepoint, median values for both parameters in all three groups were in the normal range.

**Table 3:** Hemostatic therapy administered during the study

	**FC group (n = 85)**	**FC–PCC group (n = 63)**	**FC–PCC–FFP group (n = 9)**	**p-value**
**RBC (Units)**				
ER and intra-operative	2 (0–3)	5 (3–7)	11 (8–20)	<0.0001
ICU	2 (0–3)	3 (1–5)	6.5 (5–12)	<0.0001
24h	3 (2–6)	8 (5–11)	21 (18–26)	<0.0001
**Fibrinogen concentrate (g)**				
ER and intra-operative	3 (2–3)	6 (3–7)	9 (5–16)	<0.0001
ICU	0 (0–2)	2 (0–4)	4 (0–6)	0.0006
24h	3 (2–5)	7 (5–10)	15 (9–17)	<0.0001
**PCC (Units)**				
ER and intra-operative		1200 (0–1800)	2400 (600–5100)	0.036
ICU		900 (0–1800)	1500 (0–2850)	0.34
24h		1800 (1200–2400)	4200 (3000–6150)	0.0029
**FFP (Units)**				
ER and intra-operative			0 (0–6)	
ICU			6 (0–10)	
24h			6 (6–10)	
**Platelet concentrate (Units)**				
ER and intra-operative	0 (0–0)	0 (0–0)	2 (0–2)	<0.0001
ICU	0 (0–0)	0 (0–0)	2 (1–2)	<0.0001
24h	0 (0–0)	0 (0–0)	4 (2–4)	<0.0001
**Tranexamic acid (g)**				
ER and intra-operative	0 (0–2)	0 (0–2)	0 (0–2)	0.0006
ICU	0 (0–0)	0 (0–0)	0 (0–0)	ns
24h	0 (0–2)	0 (0–2)	0 (0–2)	<0.0001

Data are presented as median (interquartile range). p-values are derived from the ANOVA or, for units of PCC, the Mann–Whitney *U* test; significance level p < 0.05.

FFP, fresh frozen plasma; ns, not significant; PCC, prothrombin complex concentrate; RBC, red blood cells.

FC group, patients receiving fibrinogen concentrate only; FC–PCC group, patients receiving fibrinogen concentrate and prothrombin complex concentrate; FC–PCC–FFP group, patients receiving fibrinogen concentrate, prothrombin complex concentrate and fresh frozen plasma.

Results are presented for the following periods of time: while patients were in the emergency room (ER) and undergoing surgery, while patients were in the intensive care unit (ICU), and the whole 24-hour period (24h) following ER admission.

The ratio of fibrinogen concentrate to RBC (g:U) for the 24-hour period after ER admission was 1.0:1.0 in the FC group, 0.9:1.0 in the FC–PCC group and 0.7:1.0 in the FC–PCC–FFP group. Massive transfusion (≥10 U RBC/24 hours) was observed in 40 patients: 7 (8%) in the FC group, 24 (38%) in the FC–PCC group and 9 (100%) in the FC–PCC–FFP group (p < 0.0001). The overall mortality rate was 19% (30 patients): 8% (7) in the FC group, 29% (18) in the FC–PCC group and 56% (5) in the FC–PCC–FFP group (statistically significant between-group difference, p = 0.0001).

## Discussion

To our knowledge this is the largest study of fibrinogen concentrate administration in major trauma patients. Coagulation therapy based on fibrinogen concentrate was largely effective for maintaining plasma fibrinogen levels and supranormal levels were not observed. In the FC and FC–PCC groups (94% of patients included in the study), median fibrinogen levels were between 1.5 and 2.0 g/L as recommended in the European trauma Guidelines upon admission to the ER [28]. Assessment upon admission to the ICU indicated that fibrinogen levels were maintained within this range between the two timepoints. Accordingly, median values for FIBTEM CA_10_ were also in the normal range at these timepoints. Only in the FC–PCC–FFP group – the most severely injured patients – were fibrinogen concentration and FIBTEM CA_10_ below these ranges (at both time points). Importantly, plasma fibrinogen levels at 24 hours were in the normal range in all groups.

The most striking finding was that the administration of fibrinogen concentrate in all three groups, either single-dose or repeated, did not lead to a surge in plasma fibrinogen levels by the end of surgery and arrival at ICU. However, one must consider that fibrinogen concentrate was given as haemostatic therapy to control bleeding and to support continuous clot formation. Ongoing consumption at the site of injury and surgery may be assumed, raising the question of how best to maintain the patient’s plasma fibrinogen level within a specific range (e.g. 1.5–2.0 g/L). Without fibrinogen concentrate treatment, a decrease in plasma fibrinogen level may be expected.

Plasma fibrinogen levels are not routinely measured at most trauma facilities worldwide and the importance of fibrinogen in major trauma patients has potentially been underestimated. Chambers et al. observed that plasma fibrinogen levels were critically low (<1 g/L) in most trauma patients treated with a massive transfusion protocol [10]. Low fibrinogen on admission was associated with poor outcomes, and has been identified as an independent predictor of mortality at 24 hours and 28 days (p < 0.001) [15]. Despite this, until now neither the optimal concentration of fibrinogen in plasma nor the ideal ratio of fibrinogen to RBCs has been established. In a dilution model, Bolliger et al. observed that clot formation was normalized as plasma fibrinogen levels approached 2.0 g/L, and the authors suggested this concentration as the minimum needed to improve clot formation [29]. Moreover, it was observed that with increasing amounts of fibrinogen the clot became more resistant to pro-fibrinolytic breakdown stimulated by tissue plasminogen activator [29]. Animal studies have revealed that hypothermia decreases fibrinogen synthesis and acidosis increases fibrinogen breakdown [30]; both of these conditions are common in trauma. Low plasma fibrinogen concentration has also been reported with hyperfibrinolysis (HF), another common occurrence in trauma [7,31-33]. This may be related to the fact that plasmin not only dissolves fibrin but (at high levels) also cleaves fibrinogen (hyperfibrinogenolysis) [31]. Data from the Clinical Randomization of an Antifibrinolytic in Significant Haemorrhage (CRASH-2) study and the more recent Military Application of Tranexamic Acid in Trauma Emergency Resuscitation (MATTERs) study highlighted the importance of early antifibrinolytic therapy [34-36]. Haemostatic therapy with either TXA or cryoprecipitate was associated with a similar reduction in mortality [35]. Importantly, a combination of both TXA and cryoprecipitate produced the best survival rate [35].

The algorithm for managing trauma-induced coagulopathy and bleeding at our centre [27] is based on first-line administration of coagulation factor concentrates. The rationale for not giving FFP first-line is that coagulation factor concentrates enable rapid and effective supplementation of specific factors, with ROTEM analysis allowing theragnostic treatment to be tailored according to the patient’s actual needs. In contrast, with allogeneics-based haemostatic therapy, several studies show that FFP transfusion may be insufficient to maintain or increase fibrinogen plasma concentration. Rourke et al. measured plasma fibrinogen concentration after every 4 units of RBC transfusion. They showed that treatment with RBC and FFP or platelets was insufficient to maintain plasma fibrinogen at 1.5 g/L. Only additional transfusion of cryoprecipitate resulted in maintenance of fibrinogen levels [15].

Fibrinogen has a half-life of 2.6–3.7 days; therefore the effect of exogenous fibrinogen concentrate may be expected to last for a week or more post-treatment [7]. Furthermore, supra-physiologic levels of fibrinogen may be expected postoperatively among trauma patients treated with fibrinogen concentrate. However, in our study, doses of 3 g, 6 g and 9 g of fibrinogen concentrate administered at ER admission and/or during surgery did not significantly increase plasma fibrinogen concentration or the viscoelastic measurements made later on at the ICU. The mechanism for this remains to be investigated, but it is likely to relate to blood loss, dilution via fluid resuscitation and consumption of the infused fibrinogen for clot formation at the wound area during and/or early after surgery. The evolution of fibrinogen levels after trauma could potentially also be influenced by increased consumption or metabolism of fibrinogen in the circulation, a shift of fibrinogen to the extravascular space, or down-regulation of endogenous fibrinogen synthesis.

All three study groups showed comparable plasma fibrinogen levels in the normal range (2.0–4.5 g/L [37,38]) during the first postoperative day. Importantly, the levels after 24 hours are also comparable with the ones reported by Schreiber et al. in trauma patients who did not receive fibrinogen concentrate [38]. In addition, these results are in agreement with data from a prospective study of trauma patients where fibrinogen concentration at 24 hours was identical in patients who received fibrinogen concentrate regardless of whether they had also received FFP [39]. Similar observations have been made in other settings – in four randomized controlled trials, 24-hour plasma fibrinogen levels and FIBTEM MCF were similar among patients treated with either fibrinogen concentrate or allogeneic blood products [40-43]. One of these studies found that shortly after fibrinogen concentrate infusion there was an increase in plasma fibrinogen levels, which was not apparent 2 hours after surgery [41].

It appears encouraging that hypercoagulability (generally defined by the surrogate marker of plasma fibrinogen concentration above the normal level) did not occur 24 hours after fibrinogen concentrate administration. This lack of a sustained increase in plasma fibrinogen levels suggests a low potential for late-onset complications, consistent with the reported low thrombogenic potential of fibrinogen concentrate [18]. Importantly, the desired effect of fibrinogen concentrate administration is to ensure continuous haemostatic capacity and to control bleeding satisfactorily, not primarily to maintain a specified fibrinogen plasma level. Well-designed prospective, controlled studies are necessary to confirm the clinical effect of fibrinogen concentrate after major trauma [44,45].

### Limitations

This was a retrospective study and potential errors are inherent in this type of analysis. There is an established treatment algorithm at our centre [27], but it cannot be guaranteed that all treatment decisions were in accordance with the algorithm. We were also not able to evaluate the effect of artificial colloids in this study. For example, hydroxyethyl starches interfere with the fibrin polymerization process, and this results in low FIBTEM clot amplitude [46].

Due to the retrospective study design, the potential side-effects of fibrinogen concentrate (e.g. thrombogenicity) could not be adequately assessed. A recent prospective randomized placebo-controlled study using fibrinogen concentrate in aortic surgery found no increase in thromboembolic complications [19]. With a coagulation concentrate-based approach to treatment, it should be kept in mind that PCC is a thrombin generating drug. In animal studies, high-dose PCC (50 U/kg bodyweight) resulted in thromboemboli and, in 44% of the animals, disseminated intravascular coagulation [47].

## Conclusions

In the current study, fibrinogen concentrate therapy maintained stable plasma fibrinogen concentrations during the initial phase of trauma care. In most patients, fibrinogen levels were within the range recommended in the European trauma guidelines and FIBTEM CA_10_ was in the normal range; the exception was the small group with most severe bleeding who received additional PCC and FFP, in whom lower levels were observed. At 24 hours, fibrinogen levels and FIBTEM CA_10_ were similar in severe trauma patients treated with fibrinogen concentrate alone, fibrinogen concentrate followed by PCC or fibrinogen concentrate followed by PCC and FFP, and within the normal range in all three groups. Further studies are warranted to elucidate the effects of fibrinogen supplementation on clinical outcomes in trauma-related bleeding.

## Abbreviations

ANOVA: Analysis of variance; aPTT: Activated partial thromboplastin time; CA10: Clot amplitude at 10 minutes’ running time; CFT: Clot formation time; CT: Clotting time; ER: Emergency room; EXTEM: Extrinsically activated coagulation test using tissue factor; FC group: Patients receiving fibrinogen concentrate only; FC–PCC group: Patients receiving fibrinogen concentrate and prothrombin complex concentrate; FC–PCC–FFP group: Patients receiving fibrinogen concentrate, prothrombin complex concentrate and fresh frozen plasma; FFP: Fresh frozen plasma; FIBTEM: Fibrin polymerization test; GCS: Glasgow coma scale; Hb: Haemoglobin; HF: Hyperfibrinolysis; ICU: Intensive care unit; ISS: Injury severity score; MCF: Maximum clot firmness; NISS: New injury severity score; PT: Prothrombin time; RBC: Red blood cell; SCT: Standard coagulation test; SD-plasma: Solvent/detergent-treated plasma; TXA: Tranexamic acid.

## Competing interests

CJS has received research support and speaker fees from CSL Behring and research support from Tem International. WV received coverage of travel costs from CSL Behring for one study group meeting. KI has received compensation for advisory board meetings sponsored by CSL Behring. MM has received consultancy fees and speaker fees from CSL Behring and Biotest. HS has received study grants and speaker fees from CSL Behring and Tem International.

## Authors’ contributions

HS and CJS conceived the study, performed data collection, analysed the data and drafted the manuscript. WV, KI, and MM contributed to the study design and drafted the manuscript. All authors have read and approved the final manuscript for publication.
